# A Fool to Do Your Dirty Work?

**DOI:** 10.1371/journal.pbio.1001859

**Published:** 2014-05-13

**Authors:** Jonathan M. Chase

**Affiliations:** Freelance Science Writer, Saint Louis, Missouri, United States of America; This is the Synopsis for PBIOLOGY-D-13-04145

**Figure:**
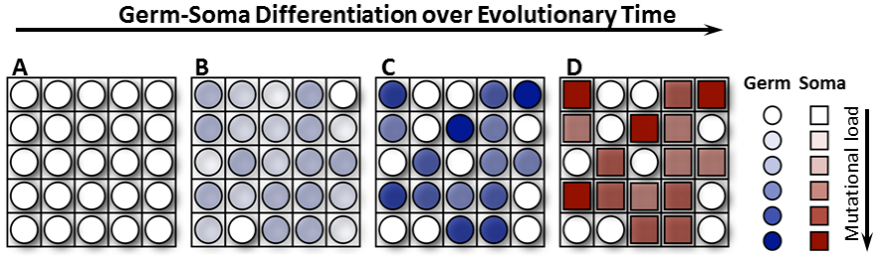
Over evolutionary time, multicells change from consisting entirely of germ cells (A) to consisting of a blend of germ and soma cells (D), where germ cells serve as propagules (founders for a new multicell) and soma cells perform the mutagenic work. (A) Germ cells do not perform mutagenic work. (B) Germs cell do perform mutagenic work. (C) A subset of germ cells performs mutagenic work. (D) Soma cells, but not germ cells, perform mutagenic work.

Much of life on Earth owes its spectacular success to a rather important evolutionary transition—from single-celled organisms to multicellularity—which has occurred independently in many lineages, enabling the differentiation of cells to perform the highly specialized functions that we see in living fungi, plants, and animals. However, whereas all clones of single-celled organisms have a relatively equal chance of dividing and propagating their genes, most multicellular organisms entrust the propagation of their genes to a few select germline cells amidst a sea of non-reproductive somatic cells. At this point, the fitness of individual cells and the fitness of the entire organism become decoupled.

Anytime complexity increases through evolution, one must ask how selection at the lower level of organization (i.e., the individual cell) doesn't disrupt the integration at higher levels of organization (i.e., a multicellular organism) by favoring selfishness. There are some general evolutionary hypotheses that have been offered to explain why and how multicellularity and the division of labor between somatic and germline cells evolved, as well as the conditions under which these developments would be expected. Clearly, organisms with differentiated cells can experience many fitness advantages, such as the ability to grow larger and exploit novel resources. And along with these advantages come costs, such as the energy and materials that must be allocated towards growth and maintenance, rather than reproduction. However, there are more subtle, but no less important, constraints on an organism's ability to acquire resources, grow, metabolize, and reproduce that might also influence the evolution of cellular differentiation.

One idea that has been suggested, but not yet fully developed, is that the evolution of multicellular organisms with separate somatic and reproductive cells might be influenced by constraints on the preservation of genetic information. Most of the “work” performed by a cell—that is, the production and use of energy—takes place in the mitochondria and chloroplasts (in eukaryotes) or across membranes (in prokaryotes). As a byproduct of this work, reactive oxygen species such as hydrogen peroxide are generated. In turn, these byproducts can create oxidative stress in a cell, one result of which can be mutations to that cell's DNA. Here, the idea of the so-called “dirty work” hypothesis is that the advent of cellular differentiation allows the organism to separate the energetically costly and potentially mutagenic processes into their somatic cells, while protecting their genomes within germline cells that need perform little work.

While this and other theories about the evolution of multicellularity and cellular differentiation are intriguing, empirical evidence is less forthcoming. Some studies in yeasts and cellular slime molds, among others, have provided a few clues. But the time scales necessary to observe and manipulate the processes driving the evolution of cellular differentiation are typically prohibitive. Unless, that is, one could reproduce the evolutionary process in a realistic, but tractable way. That is just what Goldsby et al. did in this issue of *PLOS Biology*.

To explore the role of the dirty work hypothesis in the differentiation of somatic and germline cells, Goldsby et al. performed a series of evolutionary experiments on populations of digital organisms. What's a digital organism? In this case, digital organisms have a genome that comprises a fully functional computer program. These genetic programs can process numbers that flow into and out of their habitat to perform computational logic functions (e.g., AND, NOT, XOR) which order to gain resources. These genomes mutate at some defined probability, and the organisms differentially survive and reproduce as a function of their ability to acquire resources (i.e., when enough functions are executed). With this basic framework in hand, any number of evolutionary questions can be investigated simply by defining the parameters in which the digital organisms interact.

In this case, the authors explored whether and how these simple multicellular individuals make the transition to having non-reproducing somatic cells and reproductive germline cells. They established a series of evolutionary experiments where digital organisms consisting of multiple cells performed functions to gain resources, but in the process of performing certain functions experienced mutagenic consequences (i.e., dirty work). Although the instructions for each cell in the organism were simple and identical at the start of the experiment, organisms experienced small mutations in the logic functions during replication, and some of these mutations persisted, providing the digital organisms with access to many different parts of the phenotypic space of possible functions, cell types, and efficiencies.

When the digital organisms were exposed to only low levels of mutagen while performing functions, their cells remained largely undifferentiated. That is, all cells performed some work and some reproduction. However, when the mutagenic consequence of performing certain functions was moderate, the cells of the digital organisms appeared to differentiate, yielding very high proportions of cells that performed the majority of the work, but were unable to propagate themselves (i.e., soma), and leaving other cells that performed little work but were able to propagate (i.e., germline). Finally, when performing certain functions had extremely high mutagenic effects, cells were again less differentiated, most likely because the mutagenic costs of performing this work were just too great to overcome.

In addition to establishing the dirty work hypothesis as a viable mechanism that could initiate cellular differentiation towards somatic and germline cells, the study by Goldsby et al. has a number of other implications. For example, this cellular differentiation allowed the digital organisms to exploit phenotypic niches (i.e., functions) that were unavailable to undifferentiated cells—including those that experienced higher levels of mutagens—by concentrating the costly functions in the somatic cells. However, the benefits gained by exploiting this space in terms of resource acquisition also had costs. While the original organisms were immortal, after they had evolved to be able to exploit mutagenic functions they started to show signs of aging; populations of multicellular organisms that evolved somatic cells were able to exploit more mutagenic functions and thus aged more rapidly.

While the computational organisms examined in the study by Goldsby et al. are certainly quite different from life as we usually think of it, they have many of the same basic properties as living organisms and abide by the same fundamental rules of evolution. And they shed light on the potential role of oxidative stress brought on by metabolically working cells in driving the emergence of cellular differentiation between somatic and germline cells. Finally, the essence of the dirty work hypothesis explored in this paper has widespread implications for understanding the evolution of other forms of complexity that separate functions which generate oxidative stress from those involved in replication, including the division between RNA and DNA, respectively, for metabolic work and genomic information, and the division between workers and queens in eusocial animals (e.g., some bees, wasps, and ants).

**Goldsby HJ, Knoester DB, Ofria C, Kerr B (2014) The Evolutionary Origin of Somatic Cells under the Dirty Work Hypothesis.** doi:10.1371/journal.pbio.1001858

